# Is obesity associated with depression in low- and middle-income countries? Longitudinal evidence from Indonesia

**DOI:** 10.1038/s41366-025-01757-x

**Published:** 2025-04-03

**Authors:** David Colozza, Isabella Guo, Astrid Citra Padmita, Yunita Arihandayani, Evi Firna, Mauricio Avendano

**Affiliations:** 1 Nutrition Section, UNICEF, Jakarta, Indonesia; 2 Johns Hopkins Bloomberg School of Public Health, Baltimore, MD USA; 3 Directorate of Mental Health, Ministry of Health, Jakarta, Indonesia; 4 Department of Epidemiology and Health Systems, Unisanté, Center for Primary Care and Public Health, University of Lausanne, Lausanne, Switzerland

**Keywords:** Risk factors, Epidemiology, Obesity

## Abstract

**Background:**

In high-income countries, higher body weight is associated with increased risk of depressive symptoms. However, it is unclear whether this relationship applies to low-and-middle-income countries at earlier stages of the epidemiological transition. This study uses longitudinal data to examine the relationship between body weight and depressive symptoms in Indonesia.

**Methods:**

The study employs a longitudinal sample of adolescents aged 14–19 (*N* = 3360) and adults aged ≥20 (*N* = 25,669) derived from the 2007 and 2015 Indonesia Family Life Survey. Depressive symptoms were assessed using the Centre for Epidemiologic Studies Depression ten-item scale (CES-D-10). Anthropometric measurements taken by trained nurses were used to calculate overweight/obesity (BMI ≥ 23), our outcome of interest. We use linear random and individual fixed effect models, stratified by gender and age group.

**Results:**

In random effects models, there was no association between overweight and depressive symptoms among adolescents, while overweight was associated with lower depressive symptoms among adults. These results were confirmed in fixed effect models: there was no association for adolescents (−0.32, 95% Confidence Interval [CI] −0.84, 0.21), while among adults, becoming overweight was associated with a significant reduction in depressive symptoms (−0.25, 95% CI −0.43, −0.08). There was no evidence of significant differences by sex.

**Conclusions:**

Contrary to high-income countries, we found no evidence of an association between depressive symptoms and overweight among adolescents in Indonesia, while depressive symptoms are associated with reduced risk of overweight among adults. Findings may be due to lower overweight stigma in Indonesia’s socio-cultural environment, potential depressive symptom underestimation, and a moderating role of socioeconomic status. Given the rising overweight burden in Indonesia, our results highlight the need to prioritise policies addressing structural causes rather than individual factors, in order to avoid promoting overweight stigma and safeguard mental health.

## Introduction

The global prevalence of overweight and obesity (henceforth “overweight”) has increased rapidly over the last decades in low- and middle-income countries (LMICs). High body mass index (BMI) now ranks as the fifth leading risk factor for death and disability worldwide [1]. Non-communicable diseases associated with overweight are responsible for 70% of global deaths, with three-quarters of these occurring in LMICs [2]. Concurrently, mental disorders, including depression, have emerged as significant contributors to disability-adjusted life years (DALYs) in LMICs [1]. In line with these trends, Indonesia has seen a rise in both overweight and mental health problems in recent decades. As of 2023, 37.8% of adults, 19.7% of school-age children (aged 5–12), and 14.3% of adolescents (aged 13–18) were affected by overweight or obesity [3] Meanwhile, while national survey data suggest a low prevalence of mental health disorders (2%) and depression (1.4%) among individuals aged 15 years and above [3], mental disorders, with depression at the forefront, ranked as the ninth cause of DALYs in 2019, reflecting a 73% increase compared to figures from 1990 [1]. The COVID-19 pandemic has further exacerbated mental health problems [4], and stigma surrounding mental illness persists, while access to services remains limited [5–7].

The relationship between depression and overweight is well-documented in the literature [8–11], including in children and adolescents [12, 13]. Numerous cross-sectional and longitudinal meta-analyses consistently report a positive association between depressive symptoms and overweight. This association has been linked to a broad range of biological, behavioural and psycho-social factors [14–16]. Longitudinal studies further suggest that the relationship between overweight and depression is bidirectional. On the one hand, overweight may increase the risk of depression by negatively affecting physical health, or via psycho-social mechanisms, whereby body image dissatisfaction, dieting, and weight stigma increase depression risk [16, 17]. Conversely, depression might increase the risk of overweight, through biological effects, linked for example to chronic cortisol elevation and related high-stress levels, which can promote weight gain, or indirectly, through mechanisms such as binge eating, poor adherence to weight management programmes, negative thoughts and low social support [15–17].

Much of the biomedical literature investigates the hypothesis that this bidirectional relationship may result from common underlying biological mechanisms [15, 17]. Studies looking at genetic factors suggest overlaps in brain regions responsible for appetite and mood regulation, and in genetic loci associated with both depression and BMI. Other studies point to a possible role of alterations in systems involved in homoeostatic adjustments, such as hyperactivation of the hypothalamic–pituitary–adrenal axis, leading to prolonged cortisol exposure, typically associated with both overweight and depression. Other mechanisms encompass chronic inflammation, disruptions in neuroendocrine regulators of energy metabolism, gut microbiome changes, and brain mechanisms governing appetitive and homoeostatic regulatory processes [15, 17].

A number of risk factors, including gender and socio-economic status [18, 19], have been consistently found to moderate the risk of depression in individuals with overweight. If the relationship between depression and overweight stems primarily from shared biological mechanisms, we would anticipate its presence to be nearly universal across diverse cultures and settings. Conversely, if this connection arises due to societal stigma linked to overweight, we might expect it to be weaker in regions where such stigma is less prevalent. Current research regarding the association between overweight and depression concentrates primarily on high-income countries, where overweight generally carries a significant social stigma [8, 19]. Comparatively less is known about LMICs, particularly in the context of Southeast Asia, where social perceptions and norms surrounding overweight may not carry the same degree of stigma observed in high-income countries. This could in turn be due to favourable views of overweight as an indicator of good health and higher socioeconomic status [20, 21], or to cultural factors that may discourage overt stigmatisation and discrimination against individuals with overweight [22]. Indonesia, the largest country in Southeast Asia, offers a unique context to investigate this hypothesis, as there is some evidence suggesting that overweight-related stigma may be less widespread compared to high-income countries [23, 24].

In this study, we provide novel evidence on the relationship between overweight and depression, using longitudinal data and a sample of adolescents and adults in 13 provinces across Indonesia. We use longitudinal data and fixed effect models to examine how individual-level changes in obesity relate to individual-level changes in depressive symptoms. Fixed effect models, which compare the same individuals across time points, control for time-invariant confounders such as genetic variants, shedding some light on the question of whether the association between obesity and depression may be due to unmeasured confounding. Indonesia also offers a unique context to assess whether this relationship is present in a country with less stigma associated with overweight and obesity than in high-income countries.

## Methods

### Sample

We use data from the Indonesia Family Life Survey (IFLS)^1^, an ongoing longitudinal study of socio-economic and health conditions across 13 Indonesian provinces^2^, and representative of 83% of the Indonesian population at baseline in 1993. We use specific data from the fourth and fifth waves, rolled out in 2007 and 2015, as these are the only waves with information on both depressive disorders and obesity. The sample consists of the 29,029 individuals included in the IFLS CES-D survey book at baseline. Of these, 11.6% (*N* = 3360) are adolescents aged 14–19, and 88.4% (*N* = 25,669) adults aged ≥20. At baseline, three individuals (0.01%) had missing data for CES-D scores, and 1956 (6.7%) for BMI, while at follow-up, 38.2% and 41.3% respectively were missing information for these variables (Table 1).

**Table 1:** Summary of missing data in the baseline and follow-up samples.

Variable	2007			2015		
	Missing	Non missing	% Missing	Missing	Non missing	% Missing
CES-D	3	29,026	0.01	11,090	17,939	38.2
BMI	1956	27,073	6.74	11,983	17,046	41.3
Sex	0.0	29,029	0.0	0.0	29,029	0.0
Age	0.0	29,029	0.0	0.0	29,029	0.0
Education	2136	26,893	7.36	12,126	16,903	41.8
Expenditures	22	29,007	0.08	11,122	17,907	38.3
Marital status	0.0	29,029	0.0	0.0	29,029	0.0
Urban residence	0.0	29,029	0.0	0.0	29,029	0.0

The main reason for missing data was loss to follow up in the period between the two waves considered, which spans approximately eight years. We used multiple imputation to create 20 multiply imputed data sets to use in our analyses, using the *mi* suite of commands available in Stata v. 14. We did not detect any substantial difference in results from the multiply imputed dataset (presented in the manuscript), compared to those from the unbalanced sample (including records with data for CES-D and BMI, but missing some or all controls; *N* = 27,072), and from the balanced sample (including records with data for all variables; *N* = 25,008). We report in the Supplementary Materials a comparison of results from the three samples.

### Variables

Depression was operationalised using the Centre for Epidemiological Studies Depression Scale. The scale ranges from 0 to 30 with a suggested threshold of ten indicating depressive symptoms. Considering that the CES-D has not been validated specifically for the Indonesian context against a psychiatric diagnosis, we model continuous CES-D scores as our outcome variable, as done in earlier studies [7]. The lack of validation is a potential limitation. However, previous research developing a culturally relevant scale to assess depressive symptoms in Indonesia reported a strong correlation between this contextual scale and the CES-D [25]. Additionally, other studies have found the CES-D scale, in its revised version (CESD-R), suitable for use specifically among Indonesian adolescents [26]. While these findings may not entirely eliminate potential bias, they support the appropriateness of using the CES-D scale for epidemiological purposes, as is the case in our study, if not for clinical diagnosis.

Overweight/obesity—the main independent variable in the study—was measured based on body-mass index (BMI) scores, derived from measures of height and weight included in each IFLS wave. Anthropometric measurements in IFLS were taken by trained nurses, who visited households and measured each respondent’s height and weight, alongside other biomarkers. We use BMI to create a binary variable that indicates whether an individual was affected by overweight or obesity based on the WHO Asia-Pacific cut-off for adults (BMI ≥ 23), and on gender- and age-specific cut-offs for adolescents [27]. Our decision to use a binary variable over a categorical variable distinguishing overweight from obesity was based on the small percentage of individuals with obesity (BMI ≥ 25, as per Asia-Pacific cut-off) in the adolescent age group. A small number (*N* = 12) of implausible BMI values (≥3 times below the lower boundary of the interquartile range) were assumed to be due to data entry errors and set to missing. Multiple sensitivity analyses were conducted, among others, to assess the effect of using a categorical variable for BMI (distinguishing between overweight and obesity) instead of a binary variable, and of excluding outliers. Results were robust to these different specifications (see the ‘Results’ section for additional details on the sensitivity analyses).

We conducted analyses separately for adolescents aged 14–19 and adults aged ≥20. For each, we controlled by the two other independent variables of interest, namely gender (binary variable, 1 if male 0 if female), and household per capita expenditures (as a proxy for income) (log-transformed ordinal variable indicating low, middle, and high-income), with 2015 nominal prices adjusted to real prices using the Organization for Economic Cooperation and Development Current Price Index data for Indonesia. Other control variables included marital status (binary variable, 1 if ever married, 0 if otherwise), highest level of education (no or primary education, secondary education, and tertiary education), and urban residence (1 if urban, 0 if rural).

### Statistical analyses

We first examine the baseline sample (2007) to provide descriptive sample statistics. Next, we exploit the longitudinal nature of the IFLS to understand trends in depressive symptoms across the 8-year period considered, and to study whether overweight is a predictor of depressive symptoms in the two groups considered, controlling for gender, income group, and other known confounders. We first fit a linear random effects (RE) model, controlling only for age, gender, and year (Model 1) and then a full model including all controls (Model 2). Following this, we implement fixed effects (FE) models, which exploit variation within individuals only, and assess whether within-individual changes in obesity are associated with within-individual changes in depression risk. FE models reduce potential bias due to unobserved time-invariant variables (e.g., genetics, socioeconomic background). A robust Hausman specification test suggested the use of FE models was preferable over RE models, so we base our main interpretation on results from the former.

We use individual longitudinal survey weights available in the IFLS in all analyses, which account for the sample design and attrition over the period considered and make the population of the survey waves considered representative of the Indonesian population living in the 13 provinces included at the onset of the study. In some analyses, we also include an interaction term for gender and overweight, based on available data for Indonesia indicating a higher burden of both obesity and mental health issues among adolescent girls and adult women.

## Results

Table 2 provides summary sample statistics for the sample broken down by age group (adolescents and adults) and the highest quartile of BMI. At baseline, 9.9% and adolescents and 39.8% of adults were affected by overweight. Among those in the highest quartile of BMI, a substantially higher proportion were female (70.6% among adolescents, and 69.5% among adults). For both age groups, the average CES-D scores of individuals in the highest BMI quartile were similar to those of the whole sample. Conversely, those in the highest BMI quartile had on average higher household expenditures and educational attainment.

**Table 2:** Baseline sample summary statistics.

	Tot sample	Adolescents (14–19)		Adults (≥20)	
		All	BMI p75	All	BMI p75
*N*	25,008	2823	701	22,185	5,610
Age	35.4 (SD: 14.3)	17.1 (SD: 1.4)	17.3 (SD: 1.4)	37.8 (SD: 13.5)	40 (SD: 12.1)
Female (%)	52	53.5	70.6	48.2	69.5
CES-D (continuous)	4.1 (SD: 3.5)	4.4 (SD: 3.8)	4.4 (SD: 3.6)	4 (SD: 3.5)	3.7 (SD: 3.4)
Overweight					
*BMI continuous*	22.3 (SD: 3.8)	19.8 (SD: 2.6)	23.2 (SD: 2.1)	22.5 (SD: 3.8)	27.7 (SD: 2.5)
*WHO cut-off (BMI* ≥ 23*)*	36.4	9.9	39.6	39.8	100.0
Education (%)					
*No education*	38.2	14.3	15.1	41.3	40.6
*Primary education or higher*	61.8	85.7	84.9	58.7	59.4
Urban (%)	54.1	51	52.2	54.5	62.1
Currently married (%)	71	10.3	16.8	78.8	86.6
Household expenditures					
Continuous (p/capita, IDR 10,000)	242.1 (SD: 1200)	308.8 (SD: 1580)	418.3 (SD: 2200)	233.6 (SD: 1140)	290.2 (SD: 1090)
Low (Quantile 1)	31.5	26.7	23.1	32.1	23.1
Medium (Quantile 2)	34.5	33.9	33.8	34.5	35.2
High (Quantile 3)	34	39.4	43.1	33.4	41.7

**Fig. 1:**
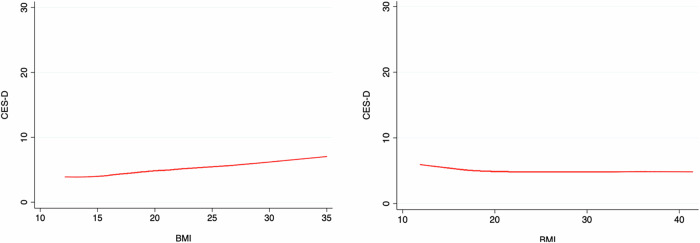
Lowess smoothing curves (bandwidth = 0.8) for CES-D and BMI for adolescents (left) and adults (right).

Figure 1 presents Lowess nonparametric curves for the relationship between CES-D scores and BMI, suggesting a positive association for adolescents (higher BMI is associated with higher CES-D scores), and a null or slightly negative association for adults.

Table 3 presents the results of RE models. There was no association between overweight and depressive symptoms among adolescents (−0.329, 95 Confidence Interval [95%CI] −0.67, 0.01), while there was a significant negative association among adults (−0.300, 95%CI −0.40, −0.20). In models that incorporated an interaction between overweight and sex, we found no evidence of differences in the association by sex. Depressive symptoms were positively associated with the highest household expenditures group among adolescents, but not among adults, and showed consistent negative associations with higher educational attainment for both age groups.

**Table 3:** Results of the longitudinal random effects regression of CES-D scores, BMI, and control variables of interest (Model 1), by age group, with and without an interaction term between gender and overweight.

	Model 1 (without interaction term)		Model 1 (with interaction term)	
	Adolescents	Adults	Adolescents	Adults
Overweight (dummy, WHO reference)	−0.329	−0.300***	−0.184	−0.282***
	[−0.67, 0.01]	[−0.40, −0.20]	[−0.61, 0.24]	[−0.40, −0.16]
Male^a^	−0.456**	−0.112*	−0.382**	−0.095
	[−0.74, −0.17]	[−0.20, −0.03]	[−0.66, −0.10]	[−0.21, 0.02]
Male*Overweight	-	-	−0.402	−0.041
	-	-	[−1.12, 0.32]	[−0.21, 0.13]
Age	0.082	−0.012***	0.082	−0.012***
	[−0.00,0.17]	[−0.02, −0.01]	[−0.00, 0.17]	[−0.02, −0.01]
Year dummy	2.705***	2.284***	2.706***	2.284***
	[2.08, 3.33]	[2.19, 2.38]	[2.08, 3.33]	[2.19, 2.38]
Expenditure Q2	−0.005	−0.150**	−0.005	−0.150**
	[−0.33, 0.32]	[−0.25, −0.05]	[−0.33, 0.32]	[−0.25, −0.05]
Expenditure Q3	0.319*	−0.1	0.319*	−0.1
	[0.00, 0.63]	[−0.21, 0.01]	[0.00, 0.63]	[−0.21, 0.01]
Ever married	−0.880***	−0.976***	−0.890***	−0.976***
	[−1.24, −0.52]	[−1.12, −0.84]	[−1.25, −0.53]	[−1.12, −0.84]
Urban residence	0.211	0.149**	0.212	0.149**
	[−0.06, 0.49]	[0.06, 0.24]	[−0.06, 0.49]	[0.06, 0.24]
Education 2 (secondary)	−0.268	−0.466***	−0.266	−0.465***
	[−0.61, 0.08]	[−0.57, −0.36]	[−0.61, 0.08]	[−0.57, −0.36]
Education 3 (tertiary)	−0.869***	−0.984***	−0.857***	−0.981***
	[−1.37, −0.37]	[−1.14, −0.83]	[−1.36, −0.36]	[−1.14, −0.83]
constant	3.425***	5.782***	3.384***	5.771***
	[1.96, 4.89]	[5.57, 5.99]	[1.92, 4.85]	[5.56, 5.98]
*N*	6720	51338	6720	51338

Statistical significance reported at *p < 0.05, **p < 0.01 and ***p < 0.001.

^a^Given the inclusion of an interaction term for overweight and gender and the modelling of the gender variable as 0 = female, 1 = male, coefficients for overweight refer to the baseline group (female).

Table 4 shows the results from FE models, which control for time-invariant confounding. We confirm the results from RE models: there is no association between overweight and depression among adolescents (−0.316, 95%CI −0.84, 0.21), but a significant negative relation remains for adults (−0.254, 95%CI −0.43, −0.08). We find no evidence of an interaction by sex, suggesting results are not significantly different between males and females. In line with the RE models, depressive symptoms were positively associated with the highest household expenditures group for adolescents, but not for adults, and negatively associated with higher educational attainment only among adults.

**Table 4:** Results of the longitudinal fixed effects regression of CES-D scores, BMI, and control variables of interest (Model 2), by age group, with and without an interaction term for gender and overweight.

	Model 2 (without interaction term)		Model 2 (with interaction term)	
	Adolescents	Adults	Adolescents	Adults
Overweight (dummy, WHO reference)	−0.316	−0.254**	−0.397	−0.236*
	[−0.84, 0.21]	[−0.43, −0.08]	[−1.04, 0.24]	[−0.45, −0.02]
Male*Overweight	-	-	0.215	−0.04
	-	-	[−0.78, 1.21]	[−0.35, 0.27]
Age	−0.634*	−0.067	−0.633*	−0.067
	[−1.14, −0.13]	[−0.16, 0.02]	[−1.13, −0.13]	[−0.16, 0.02]
Year dummy	7.668***	2.732***	7.661***	2.731***
	[4.19, 11.15]	[2.11, 3.35]	[4.18, 11.14]	[2.11, 3.35]
Expenditure Q2	0.202	−0.033	0.203	−0.033
	[−0.32, 0.72]	[−0.18, 0.11]	[−0.32, 0.73]	[−0.18, 0.11]
Expenditure Q3	0.648*	0.126	0.649*	0.126
	[0.14, 1.15]	[−0.05, 0.30]	[0.14, 1.15]	[−0.05, 0.30]
Ever married	−0.788**	−0.684***	−0.786**	−0.684***
	[−1.35, −0.22]	[−0.92, −0.45]	[−1.35, −0.22]	[−0.92, −0.45]
Urban residence	−0.125	−0.004	−0.115	−0.005
	[−0.96, 0.71]	[−0.26, 0.25]	[−0.95, 0.72]	[−0.26, 0.25]
Education 2 (secondary)	−0.273	−0.505***	−0.276	−0.505***
	[−0.89, 0.34]	[−0.73, −0.28]	[−0.89, 0.34]	[−0.73, −0.28]
Education 3 (tertiary)	−0.71	−0.943***	−0.717	−0.942***
	[−1.49, 0.07]	[−1.30, −0.58]	[−1.50, 0.06]	[−1.30, −0.58]
constant	15.335***	7.612***	15.319***	7.609***
	[6.77, 23.90]	[4.06, 11.17]	[6.75, 23.89]	[4.05, 11.16]
*N*	6720	51338	6720	51338

Statistical significance reported at *p < 0.05, **p < 0.01 and ***p < 0.001.

### Sensitivity analyses

Multiple sensitivity analyses were conducted to test the assumptions underlying our modelling strategy and ensure the robustness of our results. For the dependent variable (CES-D score), we tested the robustness of using it as a continuous variable by running alternative analyses with two dummy variables: one indicating the presence of at least one depressive symptom, and the other for the highest quartile of CES-D scores. For the main independent variable (overweight status based on BMI), we tested alternative dummy variables using different cut-offs, including the WHO global standard (BMI ≥ 25) instead of the Asia-Pacific cut-offs, and the highest BMI quartile in the sample.

Additionally, we ran analyses distinguishing between overweight and severe overweight (obesity) using WHO Asia-Pacific, global, and quartile-based cut-offs, to test the hypothesis of differential effects on mental health depending on severity. We also included BMI as a continuous variable and tested models reintegrating BMI outliers removed in the main analyses. To explore potential interactions, we tested for the hypothesis that individuals with overweight living in urban areas might be at higher risk for depressive symptoms. We also re-ran models including individuals who had been excluded due to missing data on control variables such as household expenditures (*N* = 12) and educational attainment (*N* = 2052). Overall, our results were robust across these sensitivity analyses.

Finally, we applied the same analyses to both the unbalanced sample, which included records with CES-D and BMI data but some missing control variables (*N* = 27,072), and the balanced sample, which contained complete data for all variables (*N* = 25,008). Results were consistent across the three samples. For the main variables (BMI and CES-D), there was no difference in sign or significance in the RE models. In the FE model, the association was negative and significant in the multiply imputed sample but not in the unbalanced or balanced panels; however, the direction of the association remained the same. The significance, direction, and effect sizes of control variables were generally consistent across all samples in both RE and FE models.

## Discussion

This study examined the association between depressive symptoms and overweight among Indonesian adolescents and adults. First, results from RE models suggest that, in Indonesia, living with overweight is associated with less depressive symptoms among adults, while there is no association among girls. These results are confirmed in FE models, suggesting that the association for adults cannot be explained by time-invariant confounding. Our findings contradict results from many high-income countries, and offer some support to the hypothesis that the relationship between overweight and depressive symptoms may be the result of socio-cultural factors, rather than biological mechanisms.

To shed some light on why findings for Indonesia differ from those in high-income countries, we identify three main potential mechanisms. First, cross-cultural influences related to the perception of overweight and obesity stigma might act as a context-specific moderator of the relationship between high BMI and depressive symptoms. There is evidence that cultural factors are key to influencing beliefs related to food consumption, and attitudes towards weight and health more generally [28–30]. Early accounts of body image perceptions in non-Western countries reported a possible cultural preference for overweight as a positive characteristic in low and middle-income countries at early stages of globalisation where individuals, particularly among lower-income groups, may be less exposed to global cultural norms and standards of thinness as an ideal body type [31–33]. However, evidence from a wide range of non-Western countries developed since suggests a more complicated relationship, whereby global body image standards interact with local cultural norms to delineate context-specific perceptions of ideal body image and expressions of weight stigma [33, 34]. For example, a study among Jamaican youths found no association between wealth, overweight stigma, and ideal body image [34], and studies conducted in Indonesia and Vietnam indicate positive perceptions of overweight as a sign of good health and higher socio-economic status [20, 21]. Additionally, evidence from Singapore suggest a possible role of local cultural values in restraining open stigmatisation and discrimination against people with overweight [22].

Some evidence also suggests that it is not weight status itself that affects mental health, but rather the stress of adhering to social norms and avoiding weight stigma [35–37], also among children [37, 38]. There is also evidence that being overweight is associated with discrimination in employment, education and healthcare [39]. A corollary of the context-specific nature of body image standards is that, in contexts where cultural values do not promote a body image ideal centred around thinness, and where discrimination against people with overweight is not present or not as pervasive, high BMI might not be associated with depressive symptoms. While available evidence on weight stigma among Indonesians is limited, a recent multi-country study conducted among adolescents in Brazil, South Africa and Indonesia show that Indonesian adolescents were less likely to have experienced weight stigma and to express negative perceptions of their body weight [23]. Additionally, Indonesian adolescents studied were less likely to report internalised perceptions of weight stigma, and to feel uncomfortable associating themselves with someone affected by overweight [23]. Similarly, a study of 12,000 Indonesian adults and adolescents found a positive association between overweight status and broader perceptions of happiness and quality of life [24]. These observations suggest the possible existence of a cultural environment in Indonesia that does not (yet) foster as pervasive a stigma against overweight individuals; in turn, this highlights the importance of promoting supportive environments around overweight and body image to safeguard mental health, including among young people.

A second pathway relates to the current state of global mental health care. Awareness and prioritisation of mental health issues are still low in many low and middle-income countries, reflecting in a very low availability of screening, referral, and treatment services [40–44]. Indonesia is no exception. While mental health disorders account for one quarter of the burden of disease among adolescents and young people aged 15–24 in Indonesia [45], the availability of mental health services and specialised mental healthcare professionals remains uneven across the country, with those receiving treatments for mental disorders (including depression) in the single digits [46]. Two major drivers behind this treatment gap are the lack of mental health literacy, with very few (12.7%) Indonesians aged ≥15 affected by depression seeking treatment [3], and the pervasive stigma still associated with mental health disorders [45, 46], which also results from local cultural and religious values that promote blaming individual responsibility for the development of mental disorders, thus contributing to self-stigma [45]. In combination, these factors could lead individuals to concealing mental health problems and to not reach out and seek treatment when needed, which might lead to underestimation of depressive symptoms.

A third and final pathway relates to the role of different socio-economic confounders in the association between overweight and mental health disorders. The negative relationship between lower socio-economic status and mental health disorders is well-established, including in low and middle-income countries [19] and in Indonesia specifically [7]. Moreover, available data suggest that there is a negative relationship between income and co-morbid overweight and depressive symptoms [18, 19]. However, the impact of socioeconomic status on overweight is context-specific, and may differ in Indonesia relative to high-income countries: while overweight and obesity are increasing among lower-income groups [47], available data generally suggest that the prevalence of overweight and obesity is still higher among higher income groups [47–49]. On the other hand, our results did not change in models that controlled for income or educational level.

We identify several limitations in our study. First, IFLS was not specifically designed to study overweight or mental health, so measures were not taken every wave. We were not able to directly assess the role of weight stigma in mediating the association between high BMI and depressive symptoms. Considering the consistent evidence on weight stigma associated with both mental health problems and overweight, additional research should directly measure the role of stigma. Similarly, like all self-reported health measurement tools, the CES-D is subject to potential respondent bias—particularly relevant in the context of Indonesia, where individuals may be reluctant to disclose adverse mental health conditions.

A strength of our study is that we used FE models to try to isolate the impact of overweight on depressive symptoms, controlling for time-invariant confounders. However, it is still possible that our estimates are confounded by time-varying variables, or that they also reflect the impact of depression on overweight. Finally, IFLS had high attrition rates, which may have biased our results. However, we found little evidence of differential attrition across many characteristics, and the fact that our results using MI methods did not differ from a complete case analysis renders some confidence that attrition may not be a major reason behind the lack of association between overweight and depressive symptoms in our study.

## Conclusion

Contrary to findings from high-income countries, our results suggest that, in the context of Indonesia, a large middle-income country, there is no relationship between overweight and depressive symptoms among adolescents, while overweight is associated with reduced depressive symptoms among adults. We offer three potential explanations for this finding: the absence of a socio-cultural environment in Indonesia that promotes stigma against overweight; the possible underestimation of depressive symptoms; and the role of socioeconomic status in moderating the relationship between overweight and mental health.

Our results highlight the importance of preserving and promoting supportive environments that do not encourage overweight stigma and unhealthy body image ideals, to safeguard in turn mental health, among both adolescents and adults. This is especially crucial in the context of Indonesia, considering that the rapid nutrition and epidemiological transitions currently underway are likely to increase further the burden of overweight in future decades. While it is key to develop policies and programmes to tackle overweight, efforts should be made to ensure these do not promote stigma against people with overweight. In this sense, overweight prevention strategies centred around addressing structural factors—such as effective food labelling schemes, taxation and restrictions to the sale and marketing of unhealthy foods—may be more effective in reducing the burden of this condition without increasing the risk of stigma.

## Supplementary information

Supplementary materials

## Data Availability

Data used in the study were extracted from the Indonesia Family Life Survey (IFLS), freely available upon registration from the RAND Corporation website: https://www.rand.org/well-being/social-and-behavioral-policy/data/FLS/IFLS.html.

## Footnotes

1 To date, five distinct IFLS survey waves have been rolled out in 1993, 1997, 2000, 2007, and 2015. For detailed information on the IFLS, see: https://www.rand.org/well-being/social-and-behavioral-policy/data/FLS/IFLS.html

2 Provinces included are North, West, and South Sumatra; Lampung; DKI Jakarta; West, Central Java, and East Java; DI Yogyakarta; Bali; West Nusa Tenggara; South Kalimantan; South Sulawesi.

## References

[1] Institute for Health Metrics and Evaluation (IHME). GBD compare data. 2019.

[2] WHO. Non-communicable diseases progress monitor 2022. Elsevier, 2022.

[3] Ministry of Health - RI. Indonesia Health Survey (Survei Kesehatan Indonesia, SKI) 2023. 2024.

[4] Anindyajati G, Mardiasmo DR, Sekarasih L, Susilaradeya D, Takwin B, Pelupessy DC, et al. The right to health: COVID-19 pandemic and the opportunity to transform mental health inequalities in Indonesia. Front Public Health. 2022;10:1–6. 10.3389/fpubh.2022.844656 PMC9002171 35425747

[5] Suryaputri IY, Mubasyiroh R, Idaiani S, Indrawati L. Determinants of depression in Indonesian Youth: findings from a community-based survey. J Prev Med Public Health. 2022;55:88–97. 35135052 10.3961/jpmph.21.113 PMC8841193

[6] Hartini N, Fardana NA, Ariana AD, Wardana ND. Stigma toward people with mental health problems in Indonesia. Psychol Res Behav Manag. 2018;11:535–41. 30464658 10.2147/PRBM.S175251 PMC6217178

[7] Tampubolon G, Hanandita W. Poverty and mental health in Indonesia. Soc Sci Med. 2014;106:20–27. 24524962 10.1016/j.socscimed.2014.01.012

[8] Luppino FS, De Wit LM, Bouvy PF, Stijnen T, Cuijpers P, Penninx BWJH, et al. Overweight, obesity, and depression: a systematic review and meta-analysis of longitudinal studies. Arch Gen Psychiatry. 2010;67:220–9. 20194822 10.1001/archgenpsychiatry.2010.2

[9] Iodice S, Ceresa A, Esposito CM, Mucci F, Conti DM, Pergoli L, et al. The independent role of body mass index (BMI) and severity of depressive symptoms on biological changes of women affected by overweight/obesity. Int J Environ Res Public Health. 2021;18:1–11. 10.3390/ijerph18062923 PMC8001334 33809270

[10] Stunkard AJ, Faith MS, Allison KC. Depression and obesity. Biol Psychiatry. 2003;54:330–7. 12893108 10.1016/s0006-3223(03)00608-5

[11] Blasco BV, García-Jiménez J, Bodoano I, Gutiérrez-Rojas L. Obesity and depression: Its prevalence and influence as a prognostic factor: a systematic review. Psychiatry Investig. 2020;17:715–24. 32777922 10.30773/pi.2020.0099 PMC7449839

[12] Rao WW, Zong QQ, Zhang JW, An FR, Jackson T, Ungvari GS, et al. Obesity increases the risk of depression in children and adolescents: results from a systematic review and meta-analysis. J Affect Disord. 2020;267:78–85. 32063576 10.1016/j.jad.2020.01.154

[13] Sutaria S, Devakumar D, Yasuda SS, Das S, Saxena S. Is obesity associated with depression in children? Systematic review and meta-analysis. Arch Dis Child. 2019;104:64–74. 29959128 10.1136/archdischild-2017-314608

[14] Markowitz S, Friedman MA, Arent SM. Understanding the relation between obesity and depression: Causal mechanisms and implications for treatment. Clin Psychol Sci Pract. 2008;15:1–20.

[15] Ouakinin SRS, Barreira DP, Gois CJ. Depression and obesity: integrating the role of stress, neuroendocrine dysfunction and inflammatory pathways. Front Endocrinol. 2018;9. 10.3389/fendo.2018.00431. 10.3389/fendo.2018.00431 PMC6079193 30108549

[16] Patsalos O, Keeler J, Schmidt U, Penninx BWJH, Young AH, Himmerich H. Diet, obesity, and depression: a systematic review. J Pers Med. 2021;11:1–19. 10.3390/jpm11030176 PMC7999659 33802480

[17] Milaneschi Y, Simmons WK, van Rossum EFC, Penninx BW. Depression and obesity: evidence of shared biological mechanisms. Mol Psychiatry. 2019;24:18–33. 29453413 10.1038/s41380-018-0017-5

[18] Preiss K, Brennan L, Clarke D. A systematic review of variables associated with the relationship between obesity and depression. Obes Rev. 2013;14:906–18. 23809142 10.1111/obr.12052

[19] Were JM, Hunter S, Patte KA, Leatherdale ST, Pabayo R. Income inequality and comorbid overweight/obesity and depression among a large sample of Canadian secondary school students: the mediator effect of social cohesion. SSM Popul Health 2024;28. 10.1016/j.ssmph.2024.101710. 10.1016/j.ssmph.2024.101710 PMC11417333 39319106

[20] Rachmi CN, Hunter CL, Li M, Baur LA. Perceptions of overweight by primary carers (mothers/grandmothers) of under five and elementary school-aged children in Bandung, Indonesia: a qualitative study. Int J Behav Nutr Phys Act. 2017;14:1–13. 28750666 10.1186/s12966-017-0556-1 PMC5531021

[21] Do LM, Larsson V, Tran TK, Nguyen HT, Eriksson B, Ascher H. Vietnamese mother’s conceptions of childhood overweight: findings from a qualitative study. Glob Health Action. 2016;9:1–11. 10.3402/gha.v9.30215 PMC4808081 27016327

[22] Jiang W, Tan J, Fassnacht DB. Implicit and explicit anti-fat bias among Asian females. Eat Weight Disord. 2017;22:457–65. 27206424 10.1007/s40519-016-0290-8

[23] Kataria I, Jackson-Morris A, Jewell J, Williams D, Bhandari P, Sharma D, et al. Weight stigma among adolescents in three low- and middle-income countries. J Glob Health. 2022;12:04098. 36520445 10.7189/jogh.12.04098 PMC9754065

[24] Sohn K. The fatter are happier in Indonesia. Qual Life Res. 2017;26:393–402. 27582170 10.1007/s11136-016-1403-6

[25] Widiana HS, Simpson K, Manderson L. Cultural expressions of depression and the development of the Indonesian Depression Checklist. Transcult Psychiatry. 2018;55:339–60. 29633909 10.1177/1363461518764491

[26] Tran TD, Kaligis F, Wiguna T, Willenberg L, Nguyen HTM, Luchters S, et al. Screening for depressive and anxiety disorders among adolescents in Indonesia: formal validation of the Centre for Epidemiologic Studies Depression Scale—revised and the Kessler psychological distress scale. J Affect Disord. 2019;246:189–94. 30583144 10.1016/j.jad.2018.12.042

[27] Cole TJ, Bellizzi MC, Flegal KM, Dietz WH. Establishing a standard definition for child overweight and obesity worldwide: International survey. Br Med J. 2000;320:1240–3. 10797032 10.1136/bmj.320.7244.1240 PMC27365

[28] Airhihenbuwa CO, Ford CL, Iwelunmor JI. Why Culture Matters in Health Interventions: lessons from HIV/AIDS Stigma and NCDs. Health Educ Behav. 2014;41:78–84. 23685666 10.1177/1090198113487199

[29] Panter-Brick C, Eggerman M. The field of medical anthropology in Social Science & Medicine. Soc Sci Med. 2018;196:233–9. 29137936 10.1016/j.socscimed.2017.10.033

[30] Puhl RM, Lessard LM, Pearl RL, Himmelstein MS, Foster GD. International comparisons of weight stigma: addressing a void in the field. Int J Obes. 2021;45:1976–85. 10.1038/s41366-021-00860-z 34059785

[31] Anderson-Fye EP. Body images in non-Western cultures. In: Smolak TF, Cash L (eds). *Body image: A handbook of science, practice, and prevention*. The Guilford Press, 2011.

[32] Edmonds A. *Body image in non-western societies*. Elsevier Inc., 2012 10.1016/B978-0-12-384925-0.00036-5.

[33] Eggerichs LA, Wilson OWA, Chaplin JE, Ramos Salas X. Weight stigma in Latin America, Asia, the Middle East, and Africa: a scoping review. Obes Facts. 2024;17:217–26. 38316119 10.1159/000536554 PMC11149978

[34] Anderson-Fye EP, McClure SM, Dreyer RE, Bharati A, James C. On body economics and fitting in: upward mobility and obesity stigma in Jamaica. Ethn Health. 2020;25:126–40. 29086590 10.1080/13557858.2017.1395815

[35] Brewis AA, Han SY, SturtzSreetharan CL. Weight, gender, and depressive symptoms in South Korea. Am J Hum Biol. 2017;29:1–10. 10.1002/ajhb.22972 PMC5573951 28161899

[36] Fan CW, Liu CH, Huang HH, Lin CY, Pakpour AH. Weight stigma model on quality of life among children in Hong Kong: a cross-sectional modeling study. Front Psychol. 2021;12:1–13. 10.3389/fpsyg.2021.629786 PMC8100454 33967895

[37] Lin CY, Tsai MC, Liu CH, Lin YC, Hsieh YP, Strong C. Psychological pathway from obesity-related stigma to depression via internalized stigma and self-esteem among adolescents in Taiwan. Int J Environ Res Public Health. 2019;16:1–9. 10.3390/ijerph16224410 PMC6887789 31718003

[38] Chan KL, Lee CSC, Cheng CM, Hui LY, So WT, Yu TS, et al. Investigating the relationship between weight-related self-stigma and mental health for overweight/obese children in Hong Kong. J Nerv Ment Dis. 2019;207:637–41. 31283726 10.1097/NMD.0000000000001021

[39] Clair M, Daniel C, Lamont M. Destigmatization and health: cultural constructions and the long-term reduction of stigma. Soc Sci Med. 2016;165:223–32. 27020492 10.1016/j.socscimed.2016.03.021 PMC5758051

[40] Saraceno B, van Ommeren M, Batniji R, Cohen A, Gureje O, Mahoney J, et al. Barriers to improvement of mental health services in low-income and middle-income countries. Lancet. 2007;370:1164–74. 17804061 10.1016/S0140-6736(07)61263-X

[41] Eaton J, McCay L, Semrau M, Chatterjee S, Baingana F, Araya R, et al. Scale up of services for mental health in low-income and middle-income countries. Lancet. 2011;378:1592–603. 22008429 10.1016/S0140-6736(11)60891-X

[42] Patel V, Prince M. Global Mental Health. JAMA. 2010;303:1976. 20483977 10.1001/jama.2010.616 PMC3432444

[43] Moitra M, Owens S, Hailemariam M, Wilson KS, Mensa-Kwao A, Gonese G, et al. Global mental health: where we are and where we are going. Curr Psychiatry Rep. 2023;25:301–11. 37256471 10.1007/s11920-023-01426-8 PMC10230139

[44] Kola L, Kohrt BA, Hanlon C, Naslund JA, Sikander S, Balaji M, et al. COVID-19 mental health impact and responses in low-income and middle-income countries: reimagining global mental health. Lancet Psychiatry. 2021;8:535–50. 33639109 10.1016/S2215-0366(21)00025-0 PMC9764935

[45] Brooks H, Prawira B, Windfuhr K, Irmansyah I, Lovell K, Syarif AK, et al. Mental health literacy amongst children with common mental health problems and their parents in Java, Indonesia: a qualitative study. Glob Ment Health. 2022;9:72–83. 10.1017/gmh.2022.5 PMC9806957 36618731

[46] Putri AK, Gustriawanto N, Rahapsari S, Sholikhah AR, Prabaswara S, Kusumawardhani AC, et al. Exploring the perceived challenges and support needs of Indonesian mental health stakeholders: a qualitative study. Int J Ment Health Syst. 2021;15:1–9. 34749767 10.1186/s13033-021-00504-9 PMC8573764

[47] Oddo VM, Maehara M, Rah JH. Overweight in Indonesia: an observational study of trends and risk factors among adults and children. BMJ Open. 2019;9. 10.1136/bmjopen-2019-031198. 10.1136/bmjopen-2019-031198 PMC6773342 31562157

[48] Rachmi CN, Li M, Alison Baur L. Overweight and obesity in Indonesia: prevalence and risk factors—a literature review. Public Health. 2017;147:20–9. 28404492 10.1016/j.puhe.2017.02.002

[49] Bourke EJ, Veerman JL. The potential impact of taxing sugar drinks on health inequality in Indonesia. BMJ Glob Health. 2018;3:1–8. 10.1136/bmjgh-2018-000923 PMC6267297 30555724

