# Supplementary material for: Is obesity associated with depression in low- and middle-income countries? Longitudinal evidence from Indonesia
Source: Int J Obes (Lond). 2025 Apr 3;49(6):1134–41. doi: 10.1038/s41366-025-01757-x (PMC12158782; doi:10.1038/s41366-025-01757-x)
Supplement: Supplementary file 1 — Supplementary materials [file 41366_2025_1757_MOESM1_ESM.docx]

**Supplementary materials**

**1.** Selection flowchart for the balanced sample (N=25,008) used in the sensitivity analyses.


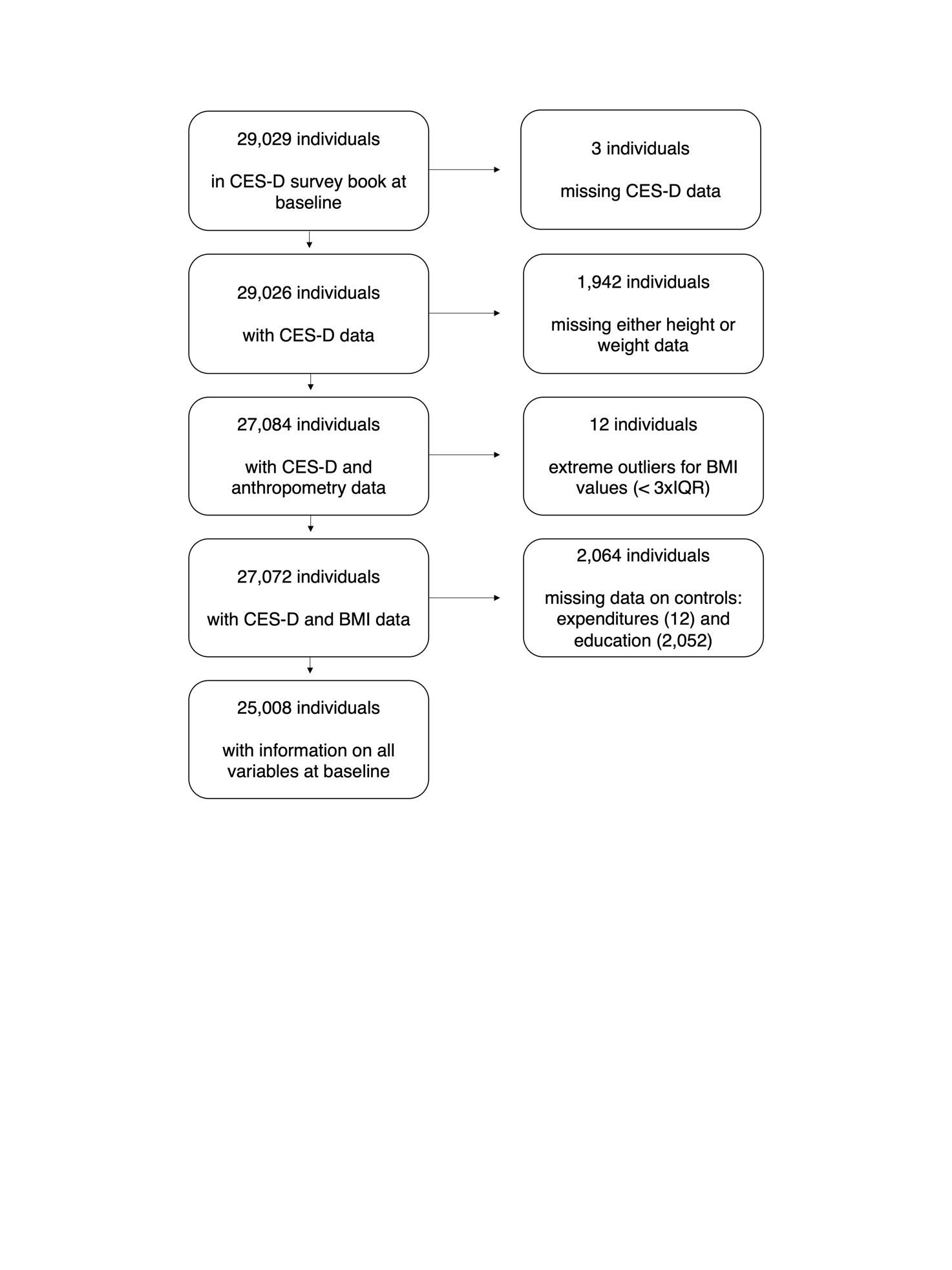


Figure A1. Selection flowchart for the balanced sample (N=25,008).

**2.** Comparison of results from the regression models on the multiply imputed sample with all individuals included in the IFLS 4 CES-D book (N=29,029), as presented in the manuscript; the unbalanced panel, including those individuals that do not have observations for the main independent variables at baseline (N=27,072); and the balanced panel, with only individuals that have observations for all variables included in the models at baseline (N=25,008).

Table A1. Comparisons for Model 1 (RE) without interactions across the multiply imputed (MI), unbalanced, and balanced samples.

|  | MI panel | | Unbalanced panel | | Balanced panel | |
| --- | --- | --- | --- | --- | --- | --- |
|  | Adolescents | Adults | Adolescents | Adults | Adolescents | Adults |
| Overweight  (dummy, WHO cut-off) | -0.329 | -0.300*** | -0.329 | -0.257*** | -0.334 | -0.265*** |
|  | [-0.67,0.01] | [-0.40,-0.20] | [-0.71,0.06] | [-0.35,-0.17] | [-0.73,0.06] | [-0.36,-0.17] |
| Male | -0.456** | -0.112* | -0.519*** | -0.051 | -0.419** | -0.051 |
|  | [-0.74,-0.17] | [-0.20,-0.03] | [-0.80,-0.24] | [-0.14,0.04] | [-0.70,-0.14] | [-0.14,0.04] |
| Age | 0.082 | -0.012*** | 0.142** | -0.015*** | 0.112* | -0.014*** |
|  | [-0.00,0.17] | [-0.02,-0.01] | [0.04,0.24] | [-0.02,-0.01] | [0.01,0.21] | [-0.02,-0.01] |
| Year dummy | 2.705*** | 2.284*** | 2.682*** | 2.233*** | 2.869*** | 2.245*** |
|  | [2.08,3.33] | [2.19,2.38] | [2.00,3.36] | [2.15,2.32] | [2.18,3.56] | [2.16,2.33] |
| Expenditure Q2 | -0.005 | -0.150** | 0.072 | -0.170** | 0.144 | -0.160** |
|  | [-0.33,0.32] | [-0.25,-0.05] | [-0.25,0.40] | [-0.27,-0.07] | [-0.18,0.47] | [-0.26,-0.06] |
| Expenditure Q3 | 0.319* | -0.1 | 0.529** | -0.109 | 0.618*** | -0.104 |
|  | [0.00,0.63] | [-0.21,0.01] | [0.19,0.87] | [-0.22,0.00] | [0.27,0.96] | [-0.22,0.01] |
| Ever married | -0.880*** | -0.976*** | -1.107*** | -0.696*** | -1.047*** | -0.711*** |
|  | [-1.24,-0.52] | [-1.12,-0.84] | [-1.52,-0.69] | [-0.81,-0.58] | [-1.47,-0.63] | [-0.83,-0.59] |
| Urban residence | 0.211 | 0.149** | 0.16 | 0.152** | 0.186 | 0.140** |
|  | [-0.06,0.49] | [0.06,0.24] | [-0.11,0.43] | [0.06,0.24] | [-0.09,0.46] | [0.05,0.23] |
| Education 2 (secondary) | -0.268 | -0.466*** | -0.241 | -0.435*** | -0.311 | -0.424*** |
|  | [-0.61,0.08] | [-0.57,-0.36] | [-0.60,0.12] | [-0.54,-0.33] | [-0.67,0.05] | [-0.53,-0.32] |
| Education 3 (tertiary) | -0.869*** | -0.984*** | -0.729* | -0.897*** | -0.721* | -0.872*** |
|  | [-1.37,-0.37] | [-1.14,-0.83] | [-1.30,-0.15] | [-1.05,-0.74] | [-1.30,-0.14] | [-1.03,-0.72] |
| constant | 3.425*** | 5.782*** | 2.302** | 5.512*** | 2.749** | 5.495*** |
|  | [1.96,4.89] | [5.57,5.99] | [0.62,3.98] | [5.32,5.71] | [1.04,4.46] | [5.30,5.69] |
| N | 6720 | 51338 | 4155 | 36868 | 3982 | 36199 |

Table A2. Comparison of Model 1 (RE) with interactions across the multiply imputed (MI), unbalanced, and balanced samples.

|  | MI panel | | Unbalanced panel | | Balanced panel | |
| --- | --- | --- | --- | --- | --- | --- |
|  | Adolescents | Adults | Adolescents | Adults | Adolescents | Adults |
| Overweight  (dummy, WHO cut-off) | -0.184 | -0.282*** | -0.08 | -0.224*** | -0.015 | -0.242*** |
|  | [-0.61,0.24] | [-0.40,-0.16] | [-0.54,0.38] | [-0.35,-0.10] | [-0.48,0.45] | [-0.36,-0.12] |
| Male | -0.382** | -0.095 | -0.413** | -0.018 | -0.284 | -0.028 |
|  | [-0.66,-0.10] | [-0.21,0.02] | [-0.70,-0.12] | [-0.14,0.10] | [-0.58,0.01] | [-0.15,0.09] |
| Male*Overweight | -0.402 | -0.041 | -0.807* | -0.075 | -1.085** | -0.052 |
|  | [-1.12,0.32] | [-0.21,0.13] | [-1.59,-0.03] | [-0.25,0.10] | [-1.88,-0.29] | [-0.23,0.12] |
| Age | 0.082 | -0.012*** | 0.143** | -0.015*** | 0.113* | -0.014*** |
|  | [-0.00,0.17] | [-0.02,-0.01] | [0.05,0.24] | [-0.02,-0.01] | [0.01,0.21] | [-0.02,-0.01] |
| Year dummy | 2.706*** | 2.284*** | 2.688*** | 2.232*** | 2.876*** | 2.244*** |
|  | [2.08,3.33] | [2.19,2.38] | [2.01,3.37] | [2.15,2.32] | [2.19,3.57] | [2.16,2.33] |
| Expenditure Q2 | -0.005 | -0.150** | 0.068 | -0.170** | 0.142 | -0.160** |
|  | [-0.33,0.32] | [-0.25,-0.05] | [-0.26,0.39] | [-0.27,-0.07] | [-0.18,0.47] | [-0.26,-0.06] |
| Expenditure Q3 | 0.319* | -0.1 | 0.523** | -0.109 | 0.614*** | -0.103 |
|  | [0.00,0.63] | [-0.21,0.01] | [0.18,0.87] | [-0.22,0.00] | [0.27,0.96] | [-0.22,0.01] |
| Ever married | -0.890*** | -0.976*** | -1.143*** | -0.696*** | -1.092*** | -0.711*** |
|  | [-1.25,-0.53] | [-1.12,-0.84] | [-1.56,-0.72] | [-0.81,-0.58] | [-1.51,-0.67] | [-0.83,-0.59] |
| Urban residence | 0.212 | 0.149** | 0.159 | 0.153** | 0.184 | 0.140** |
|  | [-0.06,0.49] | [0.06,0.24] | [-0.12,0.43] | [0.06,0.24] | [-0.09,0.46] | [0.05,0.23] |
| Education 2 (secondary) | -0.266 | -0.465*** | -0.237 | -0.433*** | -0.305 | -0.423*** |
|  | [-0.61,0.08] | [-0.57,-0.36] | [-0.60,0.12] | [-0.54,-0.33] | [-0.66,0.05] | [-0.53,-0.32] |
| Education 3 (tertiary) | -0.857*** | -0.981*** | -0.704* | -0.892*** | -0.687* | -0.869*** |
|  | [-1.36,-0.36] | [-1.14,-0.83] | [-1.28,-0.13] | [-1.05,-0.74] | [-1.27,-0.11] | [-1.02,-0.71] |
| constant | 3.384*** | 5.771*** | 2.228** | 5.491*** | 2.655** | 5.480*** |
|  | [1.92,4.85] | [5.56,5.98] | [0.55,3.91] | [5.29,5.69] | [0.95,4.36] | [5.28,5.68] |
| N | 6720 | 51338 | 4155 | 36868 | 3982 | 36199 |

Table A3. Comparison of Model 2 (FE) without interactions across the multiply imputed (MI), unbalanced, and balanced samples.

|  | MI panel | | Unbalanced panel | | Balanced panel | |
| --- | --- | --- | --- | --- | --- | --- |
|  | Adolescents | Adults | Adolescents | Adults | Adolescents | Adults |
| Overweight  (dummy, WHO cut-off) | -0.316 | -0.254** | -0.263 | -0.134 | -0.263 | -0.134 |
|  | [-0.84,0.21] | [-0.43,-0.08] | [-0.94,0.42] | [-0.33,0.07] | [-0.94,0.42] | [-0.33,0.07] |
| Age | -0.634* | -0.067 | -0.564* | -0.125* | -0.564* | -0.125* |
|  | [-1.14,-0.13] | [-0.16,0.02] | [-1.08,-0.05] | [-0.23,-0.02] | [-1.08,-0.05] | [-0.23,-0.02] |
| Year dummy | 7.668*** | 2.732*** | 7.453*** | 3.050*** | 7.453*** | 3.050*** |
|  | [4.19,11.15] | [2.11,3.35] | [3.93,10.98] | [2.31,3.79] | [3.93,10.98] | [2.31,3.79] |
| Expenditure Q2 | 0.202 | -0.033 | 0.465 | 0.107 | 0.465 | 0.107 |
|  | [-0.32,0.72] | [-0.18,0.11] | [-0.19,1.12] | [-0.06,0.27] | [-0.19,1.12] | [-0.06,0.27] |
| Expenditure Q3 | 0.648* | 0.126 | 1.210*** | 0.386*** | 1.210*** | 0.386*** |
|  | [0.14,1.15] | [-0.05,0.30] | [0.55,1.87] | [0.20,0.58] | [0.55,1.87] | [0.20,0.58] |
| Ever married | -0.788** | -0.684*** | -1.029** | -0.042 | -1.029** | -0.042 |
|  | [-1.35,-0.22] | [-0.92,-0.45] | [-1.75,-0.30] | [-0.33,0.25] | [-1.75,-0.30] | [-0.33,0.25] |
| Urban residence | -0.125 | -0.004 | -0.22 | 0.053 | -0.22 | 0.053 |
|  | [-0.96,0.71] | [-0.26,0.25] | [-1.09,0.65] | [-0.23,0.33] | [-1.09,0.65] | [-0.23,0.33] |
| Education 2 (secondary) | -0.273 | -0.505*** | 1.870* | 0.2 | 1.870* | 0.2 |
|  | [-0.89,0.34] | [-0.73,-0.28] | [0.21,3.53] | [-0.22,0.62] | [0.21,3.53] | [-0.22,0.62] |
| Education 3 (tertiary) | -0.71 | -0.943*** | 2.168* | 0.753* | 2.168* | 0.753* |
|  | [-1.49,0.07] | [-1.30,-0.58] | [0.34,4.00] | [0.11,1.39] | [0.34,4.00] | [0.11,1.39] |
| constant | 15.335*** | 7.612*** | 11.946** | 8.362*** | 11.952** | 8.356*** |
|  | [6.77,23.90] | [4.06,11.17] | [3.22,20.67] | [4.29,12.44] | [3.22,20.68] | [4.29,12.42] |
| N | 6720 | 51338 | 4155 | 36868 | 3982 | 36199 |

Table A4. Comparison of Model 2 (FE) with interactions across the multiply imputed (MI), unbalanced, and balanced samples.

|  | MI panel | | Unbalanced panel | | Balanced panel | |
| --- | --- | --- | --- | --- | --- | --- |
|  | Adolescents | Adults | Adolescents | Adults | Adolescents | Adults |
| Overweight  (dummy, WHO reference) | -0.397 | -0.236* | -0.366 | -0.132 | -0.366 | -0.132 |
|  | [-1.04,0.24] | [-0.45,-0.02] | [-1.19,0.46] | [-0.39,0.12] | [-1.19,0.46] | [-0.39,0.12] |
| Male*Overweight | 0.215 | -0.04 | 0.319 | -0.003 | 0.319 | -0.003 |
|  | [-0.78,1.21] | [-0.35,0.27] | [-0.91,1.55] | [-0.38,0.38] | [-0.91,1.55] | [-0.38,0.38] |
| Age | -0.633* | -0.067 | -0.563* | -0.125* | -0.563* | -0.125* |
|  | [-1.13,-0.13] | [-0.16,0.02] | [-1.08,-0.05] | [-0.23,-0.02] | [-1.08,-0.05] | [-0.23,-0.02] |
| Year dummy | 7.661*** | 2.731*** | 7.445*** | 3.050*** | 7.445*** | 3.050*** |
|  | [4.18,11.14] | [2.11,3.35] | [3.92,10.97] | [2.31,3.79] | [3.92,10.97] | [2.31,3.79] |
| Expenditure Q2 | 0.203 | -0.033 | 0.472 | 0.106 | 0.472 | 0.106 |
|  | [-0.32,0.73] | [-0.18,0.11] | [-0.18,1.12] | [-0.06,0.27] | [-0.18,1.12] | [-0.06,0.27] |
| Expenditure Q3 | 0.649* | 0.126 | 1.222*** | 0.386*** | 1.222*** | 0.386*** |
|  | [0.14,1.15] | [-0.05,0.30] | [0.56,1.89] | [0.20,0.58] | [0.56,1.89] | [0.20,0.58] |
| Ever married | -0.786** | -0.684*** | -1.016** | -0.042 | -1.016** | -0.042 |
|  | [-1.35,-0.22] | [-0.92,-0.45] | [-1.74,-0.29] | [-0.33,0.25] | [-1.74,-0.29] | [-0.33,0.25] |
| Urban residence | -0.115 | -0.005 | -0.204 | 0.053 | -0.204 | 0.053 |
|  | [-0.95,0.72] | [-0.26,0.25] | [-1.08,0.68] | [-0.23,0.33] | [-1.08,0.68] | [-0.23,0.33] |
| Education 2 (secondary) | -0.276 | -0.505*** | 1.861* | 0.2 | 1.861* | 0.2 |
|  | [-0.89,0.34] | [-0.73,-0.28] | [0.20,3.52] | [-0.22,0.62] | [0.20,3.52] | [-0.22,0.62] |
| Education 3 (tertiary) | -0.717 | -0.942*** | 2.154* | 0.753* | 2.154* | 0.753* |
|  | [-1.50,0.06] | [-1.30,-0.58] | [0.32,3.98] | [0.11,1.39] | [0.32,3.98] | [0.11,1.39] |
| constant | 15.319*** | 7.609*** | 11.934** | 8.362*** | 11.940** | 8.356*** |
|  | [6.75,23.89] | [4.05,11.16] | [3.21,20.66] | [4.29,12.44] | [3.21,20.67] | [4.29,12.42] |
| N | 6720 | 51338 | 4155 | 36868 | 3982 | 36199 |
